# The chromatin reader ZMYND8 recruits the NuRD component GATAD2A through its MYND domain to regulate MAPT213 long noncoding RNA transcription

**DOI:** 10.1016/j.jbc.2026.111463

**Published:** 2026-04-16

**Authors:** Dushyant Kumar Srivastava, Sandhik Nandi, Avradeep Karmakar, Chandrima Das, Siddhartha Roy

**Affiliations:** 1 Structural Biology and Bio-Informatics Division, Council of Scientific & Industrial Research-Indian Institute of Chemical Biology, Kolkata, India; 2 Biophysics & Structural Genomics Division, Saha Institute of Nuclear Physics, Kolkata, India; 3 Homi Bhabha National Institute, Mumbai, India; 4 Academy of Scientific and Innovative Research (AcSIR), Ghaziabad, Uttar Pradesh, India

**Keywords:** chromatin, MAPT, MYND domain, structural biology, transcription regulation, ZMYND8

## Abstract

The zinc finger MYND-type containing eight protein (ZMYND8) is a chromatin reader that regulates neuronal gene expression by controlling the microtubule-associated protein tau (MAPT) locus. Here, we investigate how ZMYND8 regulates expression of the long non-coding RNA MAPT213 through its interaction with GATA zinc finger domain containing 2A (GATAD2A), a component of the Nucleosome Remodelling and Deacetylase complex. ZMYND8 exhibits opposite regulatory effects on protein-coding MAPT and non-coding MAPT213 transcripts in a manner dependent on its MYND domain, promoting MAPT expression while suppressing MAPT213 levels. Chromatin immunoprecipitation experiments demonstrated that ZMYND8 specifically recruits GATAD2A to the MAPT213 internal regulatory region, establishing a direct link between protein binding and transcriptional control. We determined the crystal structure of the ZMYND8 coiled-coil MYND domain at high resolution, revealing a homodimeric architecture. The MYND domain specifically recognizes GATAD2A through direct interaction with proline-rich motifs in GATAD2A's central region. Structure-function analysis identified critical binding interface residues, while quantitative measurements revealed moderate-affinity interactions enhanced through multivalent binding mechanisms. These findings establish the molecular basis for ZMYND8-mediated recruitment of chromatin remodeling complexes to specific genomic loci and provide a structural framework for understanding transcriptional regulation of MAPT213.

Transcriptional regulation is orchestrated by multiple factors, including transcription factors, chromatin modifiers, and non-coding RNAs. Among these regulators, Zinc Finger MYND-Type Containing 8 (ZMYND8) has emerged as a crucial player in neuronal differentiation (1, 2, 3), DNA damage response (4, 5, 6, 7), and cancer progression (4, 8, 9, 10, 11, 12). ZMYND8, also known as RACK7 or PRKCBP1, is a multidomain protein belonging to the ZMYND family, characterized by the presence of a Myeloid, Nervy, and DEAF-1 (MYND) domain.

The molecular architecture of ZMYND8 reflects its dual function as both a chromatin reader and a protein interaction hub (Fig. 1*A*). The N-terminal region contains a tandem array of chromatin-binding domains—Plant Homeodomain (PHD), bromodomain, and PWWP (Pro-Trp-Trp-Pro) modules—that collectively recognize specific histone modification patterns (7, 8). The PHD finger binds methylated lysine residues on histone tails, the bromodomain recognizes acetylated lysines, and the PWWP domain binds methylated H3K36, a mark associated with active transcription (7). The C-terminal region contains a coiled-coil (CC) domain followed by the signature MYND domain, a conserved zinc finger motif known to mediate protein-protein interactions (13, 14, 15, 16). This domain is critical for ZMYND8's function in transcriptional regulation and other cellular processes.

**Figure 1:**
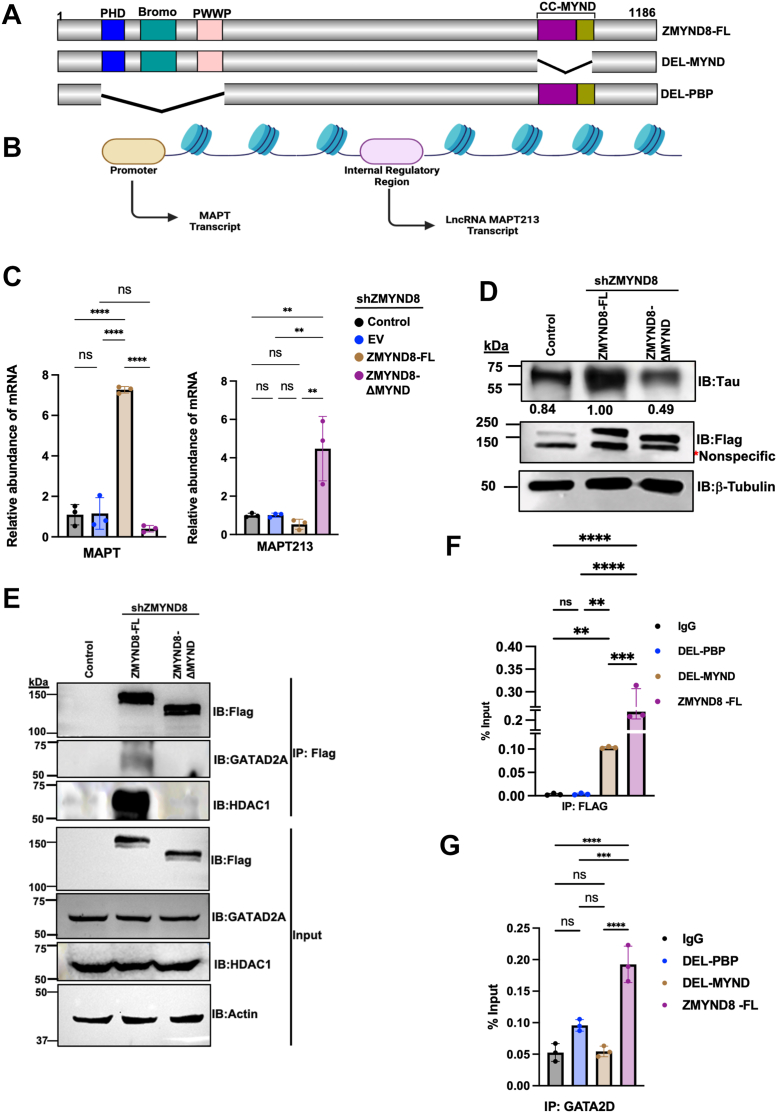
**ZMYND8 regulates MAPT and lncRNA MAPT213 expression through MYND domain-dependent recruitment of GATAD2A.***A*, schematic representation of ZMYND8 domain organization showing N-terminal Plant Homeodomain, Bromodomain (Bromo), and PWWP domain, followed by a CC and C-terminal MYND domain. Numbers indicate amino acid positions. *B*, genomic organization of the MAPT locus highlighting the promoter region and internal regulatory region from which lncRNA MAPT213 is transcribed. *C*, quantitative RT-PCR analysis of MAPT mRNA (*left panel*) and MAPT213 lncRNA (*right panel*) expression in SH-SY5Y cells (with stable knockdown of endogenous ZMYND8) transfected with FLAG-ZMYND8-FL, or FLAG-ZMYND8-ΔMYND constructs. One-way ANOVA was performed to analyze the *p*-value significance (∗*p*< 0.05; ∗∗*p*< 0.01; ∗∗∗*p*< 0.001; ns, nonsignificant (*p*> 0.05)) for the statistical analyzes, the error bar represents the SD. Each data point representing independent biological replicate (n = 3). *D*, Western blot analysis of Tau protein expression in control and transfected cells. ZMYND8 constructs were detected with anti-FLAG antibody. β-tubulin served as loading control. Numbers below Tau blot indicate densitometric quantification relative to control (set to 1.00). *E*, coimmunoprecipitation analysis showing interaction between FLAG-tagged ZMYND8 constructs and endogenous GATAD2A. Input lysates (*bottom panels*) and FLAG immunoprecipitates (*top panels*) were analyzed by western blotting with indicated antibodies. *F*, control Flag ChIP to show recruitment of Flag-ZMYND8-FL and Flag-ZMYND8-ΔMYND at the internal regulatory region. One-way ANOVA was performed to analyze the *p*-value significance (∗*p*< 0.05; ∗∗*p*< 0.01; ∗∗∗*p*< 0.001; ns, nonsignificant (*p*> 0.05)) for the statistical analyzes, the *error bar* represents the SD. Each data point representing independent biological replicate (n = 3). *G*, GATAD2A recruitment to the MAPT213 internal regulatory region is dependent on the MYND domain of ZMYND8 as observed by ChIP. One-way ANOVA was performed to analyze the *p*-value significance (∗*p*< 0.05; ∗∗*p*< 0.01; ∗∗∗*p*< 0.001; ns, nonsignificant (*p*> 0.05)) for the statistical analyzes, the *error bar* represents the SD. Each data point representing independent biological replicate (n = 3). CC, coiled-coil; ZMYND8, zinc finger MYND-type containing eight protein; ZMYND8-FL, full-length ZMYND8.

ZMYND8 has been implicated in diverse cellular functions (3, 17, 18, 19, 20, 21). In DNA damage response, ZMYND8 promotes homologous recombination repair by facilitating recruitment of the Nucleosome Remodeling Deacetylase (NuRD) complex to DNA double-strand breaks (6, 22, 23, 24). ZMYND8 also functions as a reader of enhancer-associated histone modifications and regulates enhancer-mediated gene expression, with implications for cancer progression and metastasis (25, 26, 27). In neuronal differentiation, ZMYND8 regulates expression of genes involved in neurite outgrowth and synaptic plasticity (1).

One key interaction partner of ZMYND8 is GATA Zinc Finger Domain Containing 2A (GATAD2A), also known as p66α, a component of the NuRD complex. GATAD2A is a transcriptional repressor that plays crucial roles in embryonic development, cellular differentiation, and gene silencing (28, 29, 30). It contains two conserved regions (CR1 and CR2) and a GATA-type zinc finger domain (28, 29). The CR2 domain contains multiple PPPLΦ motifs (where Φ represents a hydrophobic residue) that mediate interactions with other regulatory proteins. The NuRD complex couples histone deacetylation and nucleosome remodeling activities (31), playing critical roles in transcriptional repression, heterochromatin formation, and genomic stability. The interaction between ZMYND8 and GATAD2A suggests a mechanism by which ZMYND8 recruits chromatin-modifying activities to specific genomic loci, leading to local changes in chromatin structure and transcriptional repression. The specificity of this regulation is likely conferred by the chromatin-reading domains of ZMYND8, which recognize specific histone modifications associated with particular chromatin states.

Recent studies have highlighted ZMYND8's role in regulating genes critical for neuronal differentiation (1). Among these targets, the Microtubule Associated Protein Tau (MAPT) gene has drawn particular attention due to its importance in neurodegenerative disorders such as Alzheimer's disease. A newly identified transcript, MAPT213, has been characterized as a long non-coding RNA (lncRNA) with potential regulatory functions. The presence of an intra-gene regulatory region within MAPT213 suggests a complex regulatory mechanism governing its expression. Understanding how ZMYND8 regulates MAPT213 is important given tau's critical role in neurodegenerative disease, and represents an excellent model system for elucidating MYND domain function in gene-specific regulation.

In this study, we investigate the molecular mechanisms underlying ZMYND8-mediated regulation of MAPT213 expression. We solved the crystal structure of ZMYND8's coiled coil MYND (CC-MYND) domain at 2.29 Å resolutions, revealing a dimeric arrangement with potential for higher-order oligomerization. The MYND domain was found to be critical for ZMYND8's regulatory function, as deletion of this domain abrogated its ability to modulate MAPT and MAPT213 expression. Through biochemical and biophysical approaches, we identified a specific interaction between ZMYND8's MYND domain and GATAD2A's PPPLΦ motifs. Molecular docking, mutational analyses, and isothermal titration calorimetry (ITC) experiments pinpointed key residues in ZMYND8's MYND domain that mediate this interaction. Our findings provide structural and mechanistic insights into ZMYND8-mediated recruitment of chromatin remodeling complexes to specific genomic loci.

## Results

### The MYND domain of ZMYND8 is essential for transcriptional regulation of MAPT and long non-coding RNA MAPT213

The transcriptional regulator ZMYND8 contains multiple functional domains, including N-terminal PHD, bromodomain, and PWWP domains that facilitate chromatin binding, and a C-terminal MYND domain preceded by a CC region (Fig. 1*A*). Given that MYND domains typically mediate protein-protein interactions, we investigated whether this domain is required for ZMYND8-dependent regulation of MAPT gene expression. We focused our analysis on the MAPT gene locus, which contains both protein-coding transcripts and a recently identified long non-coding RNA, MAPT213. The MAPT213 transcript originates from an internal regulatory region within the MAPT gene that does not encode protein (Fig. 1*B*). To assess the functional role of the MYND domain, we generated three FLAG-tagged constructs for cellular studies: full-length ZMYND8 (ZMYND8-FL), a MYND domain-deleted (ZMYND8-ΔMYND), and a construct lacking the N-terminal chromatin-reading domains (ZMYND8-ΔPBP, where PBP refers to the deleted PHD-Bromodomain-PWWP domains) and transfected them into SH-SY5Y neuroblastoma cells.

Quantitative RT-PCR analysis revealed that ZMYND8-FL overexpression significantly increased MAPT mRNA levels compared to control cells, whereas ZMYND8-ΔMYND failed to enhance MAPT transcription (Fig. 1*C*; left panel). Suggesting that the MYND domain is crucial for ZMYND8-mediated regulation of MAPT transcripts. Interestingly, expression of LncRNA MAPT213 transcript shows reduced levels when ZMYND8-FL is overexpressed, and the level goes up upon overexpression of ZMYND8-ΔMYND construct (Fig. 1*C*; right panel). This inverse relationship suggests that ZMYND8 differentially regulates protein-coding MAPT and non-coding MAPT213 transcripts in a MYND domain-dependent manner. Western blot analysis confirmed that the transcriptional changes translated to protein level differences. ZMYND8-FL overexpression substantially increased tau protein expression, whereas ZMYND8-ΔMYND overexpression reduced tau levels compared to control (Fig. 1*D*), consistent with the mRNA expression patterns.

To elucidate the molecular mechanism underlying ZMYND8-mediated regulation of MAPT213, we sought to identify MYND domain-dependent binding partners that could mediate transcriptional repression. We focused on GATAD2A (also known as p66α) as it contains multiple PPPLΦ motifs (where Φ represents a hydrophobic residue) in its central region, and MYND domains in other proteins, such as CBFA2T1 and BS69, have been shown to specifically recognize proline-rich PPPLΦ or PXLXP motifs (13, 14). This suggested that GATAD2A could be a direct MYND domain binding partner.

We performed co-immunoprecipitation experiments to test whether ZMYND8 interacts with GATAD2A in a MYND domain-dependent manner. As shown in Figure 1*E*, ZMYND8-FL efficiently co-immunoprecipitated with endogenous GATAD2A. Importantly, this interaction was strictly dependent on the MYND domain, as ZMYND8-ΔMYND showed markedly reduced association with GATAD2A compared to ZMYND8-FL. Western blot analysis of both input lysates and FLAG immunoprecipitates with anti-FLAG antibody confirmed equal IP efficiency and comparable expression levels of full-length (FL) and ΔMYND constructs across samples, validating the specificity of the observed interaction differences.

To determine whether this interaction has functional significance at the chromatin level, we conducted chromatin immunoprecipitation (ChIP) assays. FLAG-ChIP experiments demonstrated that both ZMYND8-FL and ZMYND8-ΔMYND were recruited to the MAPT213 internal regulatory region, indicating that the MYND domain is not required for chromatin binding *per se* (Fig. 1*F*). However, GATAD2A recruitment to this locus was strictly MYND domain-dependent: cells expressing ZMYND8-FL showed robust GATAD2A occupancy at the MAPT213 regulatory region, whereas ZMYND8-ΔMYND-expressing cells exhibited significantly reduced GATAD2A binding (Fig. 1*G*). As negative controls, we assessed ZMYND8 binding to a region approximately 10 kb upstream of MAPT213, which showed minimal enrichment for all constructs, demonstrating binding specificity (Fig. S2 and Table S1). These findings demonstrate that ZMYND8 regulates lncRNA MAPT213 expression through a mechanism requiring its MYND domain-mediated interaction with GATAD2A.

### Crystal structure of the ZMYND8 coiled-coil MYND domain reveals a modular L-shaped architecture

**Figure 2:**
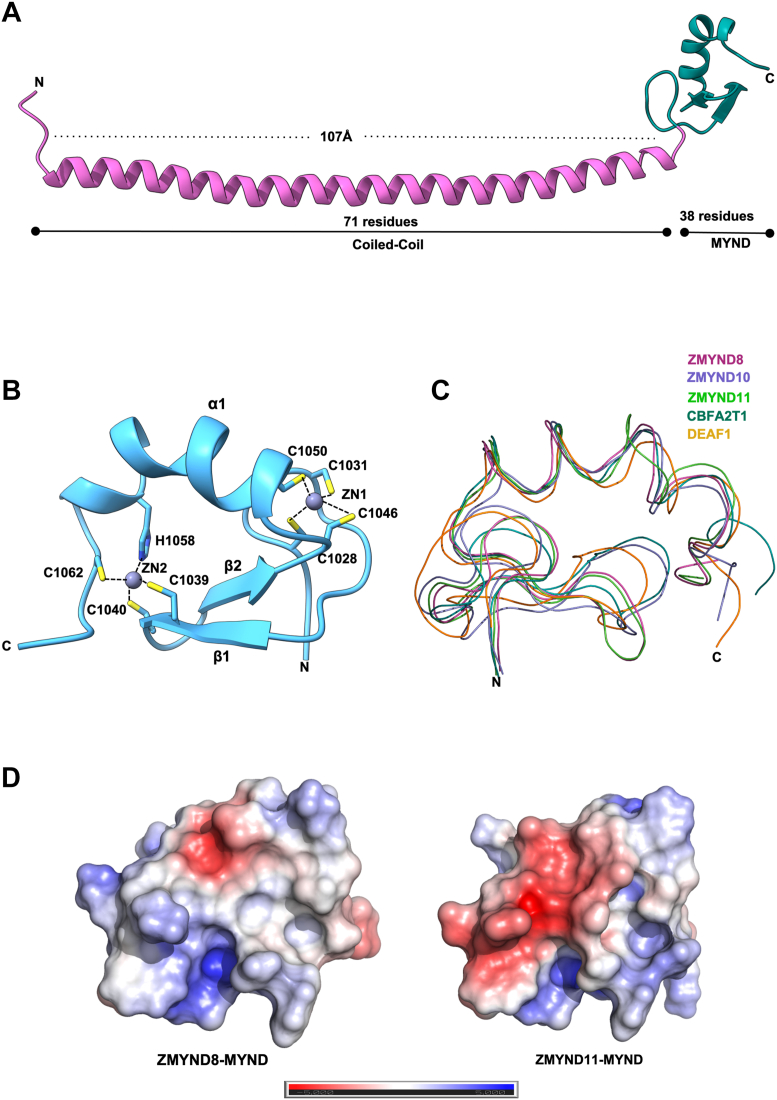
**Crystal structure of ZMYND8 CC MYND domain.***A*, ribbon representation of the ZMYND8 CC-MYND crystal structure (2.29 Å resolution) showing the L-shaped modular organization. The N-terminal CC region (*pink*, 71 residues, ∼107 Å length) is positioned perpendicular to the C-terminal MYND domain (*cyan*, 38 residues). *B*, detailed view of the MYND domain structure highlighting the zinc coordination sites. Two zinc atoms (*blue spheres*) are coordinated by conserved cysteine and histidine residues as indicated. Zn1 exhibits tetrahedral coordination by four cysteine residues (Cys1028, Cys1031, Cys1046, Cys1050), while Zn2 is coordinated by three cysteines (Cys1039, Cys1040, Cys1062) and one histidine (His1058). Secondary structure elements (β1, β2, α1) are labeled. *C*, structural superposition of MYND domains from ZMYND8 (*cyan*), ZMYND10 (*yellow*), ZMYND11 (*green*), CBFA2T1 (*orange*), and DEAF1 (*purple*) demonstrating conservation of the core fold across family members. *D*, electrostatic surface potential comparison between ZMYND8 (*left*) and ZMYND11 (*right*) MYND domains. Surface coloring represents charge distribution: *blue* (*positive*), *red* (*negative*), and *white* (*neutral*). All structural representations were generated using PyMOL, and electrostatic potentials were calculated using Adaptive Poisson-Boltzmann Solver. CC, coiled-coil; CC-MYND, coiled coil MYND; ZMYND8, zinc finger MYND-type containing eight protein; ZMYND8-FL, full-length ZMYND8.

Given the critical functional role of the MYND domain in ZMYND8-mediated transcriptional regulation, we sought to determine its high-resolution structure to provide mechanistic insights into its protein-protein interaction capabilities. We successfully crystallized and solved the structure of the ZMYND8 CC MYND domain construct (residues 951–1076) to 2.29 Å resolution using X-ray crystallography (Table 1). The crystal structure reveals a distinctive modular architecture consisting of two distinct structural elements organized in an L-shaped configuration (Fig. 2*A*). The N-terminal region forms an elongated α-helical CC domain (residues 951–1021) that extends approximately 107 Å in length and contains 71 amino acid residues. This extended helical region is oriented perpendicular to the C-terminal MYND domain (residues 1022–1076), which comprises 38 residues. The MYND domain is composed of two antiparallel β-sheets (β1 and β2) followed by a C-terminal α-helix (α1) (Fig. 2*B*). A closer examination of the MYND domain structure reveals its characteristic zinc-binding motif. The domain coordinates two zinc atoms through a conserved arrangement of cysteine and histidine residues that is characteristic of MYND domain family members. The first zinc atom (Zn1) exhibits tetrahedral coordination geometry through four cysteine residues: Cys1028, Cys1031, Cys1046, and Cys1050. The second zinc atom (Zn2) displays a slightly different coordination environment, being coordinated by three cysteine residues (Cys1039, Cys1040, and Cys1062) and one histidine residue (His1058) This zinc-binding arrangement is crucial for maintaining the structural integrity and function of the MYND domain.

**Table 1:** Data collection and refinement statistics

Protein	ZMYND8 CC-MYND domain
Data collection	
Space group	P 21 21 21
Unit cell dimension	
a, b, c (Å)	63.70, 71.19, 230.27
α, β, γ (°)	90, 90, 90
Wavelength (Å)	1.5406
Resolution (Å)	38.67–2.29 (2.34–2.29)
Total no. reflections	669,899 (30,409)
Unique no. reflections	90,783 (5091)
Multiplicitys	7.4 (6.0)
Completeness (%)	99.58 (95.65)
Mean I/sigma(I)	12.16 (1.25)
R-merge	0.1255 (1.21)
R-meas	0.1349 (1.322)
R-pim	0.04931 (0.522)
CC1/2	0.998 (0.419)
Refinement	
No. of reflection	47,990 (2659)
Reflections used for R-free	2399 (133)
R_work_/R_free_ (%)	24.2/27.3 (35.4/38.8)
No. atoms/*B*-factor (Å^2^)	
Protein	3786/66.8
ligands	34/56.7
solvent	290/50.1
Root-mean-square deviations	
Bond lengths (Å)	0.009
Bond angle (°)	0.94
Ramachandran statistics	
Favored (%)	97.07
Allowed (%)	2.71
Outliers (%)	0.23
PDB entry	9WV4

Statistics for the highest-resolution shell are shown in parentheses.

To assess the structural conservation of the MYND domain across different proteins, we performed a structural superposition of the ZMYND8 MYND domain with those from related proteins: ZMYND10(PDB ID 2DAN), ZMYND11 (PDB ID 5HDA), CBFA2T1 (PDB ID 2DJ8), and DEAF1(PDB ID 2JW6) (Fig. 2*C*). Despite originating from proteins with diverse cellular functions, all MYND domains exhibit remarkable structural conservation in their core fold, with root-mean-square deviations typically below 2 Å for the zinc-coordinating framework and secondary structure elements. This conservation underscores the importance of the MYND domain as a modular protein interaction motif in various cellular processes.

Next, we analyzed the electrostatic surface properties of the ZMYND8 MYND domain and compared it to that of ZMYND11(32). The electrostatic surface potential mapping demonstrates distinct charge distributions despite their structural similarity. The ZMYND8 MYND domain exhibits a unique arrangement of positively charged (blue), negatively charged (red), and neutral (white) surface regions that differ markedly from the ZMYND11 pattern (Fig. 2*D*).

Overall, our structural analysis of the ZMYND8 CC-MYND region reveals a modular architecture with a CC region followed by a zinc-coordinating MYND domain. The MYND domain shows structural conservation with related proteins but exhibits unique electrostatic surface properties. The distinct surface properties of the ZMYND8 MYND domain, including its unique electrostatic potential distribution, could contribute to its specific binding partners and target gene selectivity. However, further studies are needed to directly test this hypothesis.

### ZMYND8 CC-MYND forms stable homodimers through MYND domain-mediated interactions

**Figure 3:**
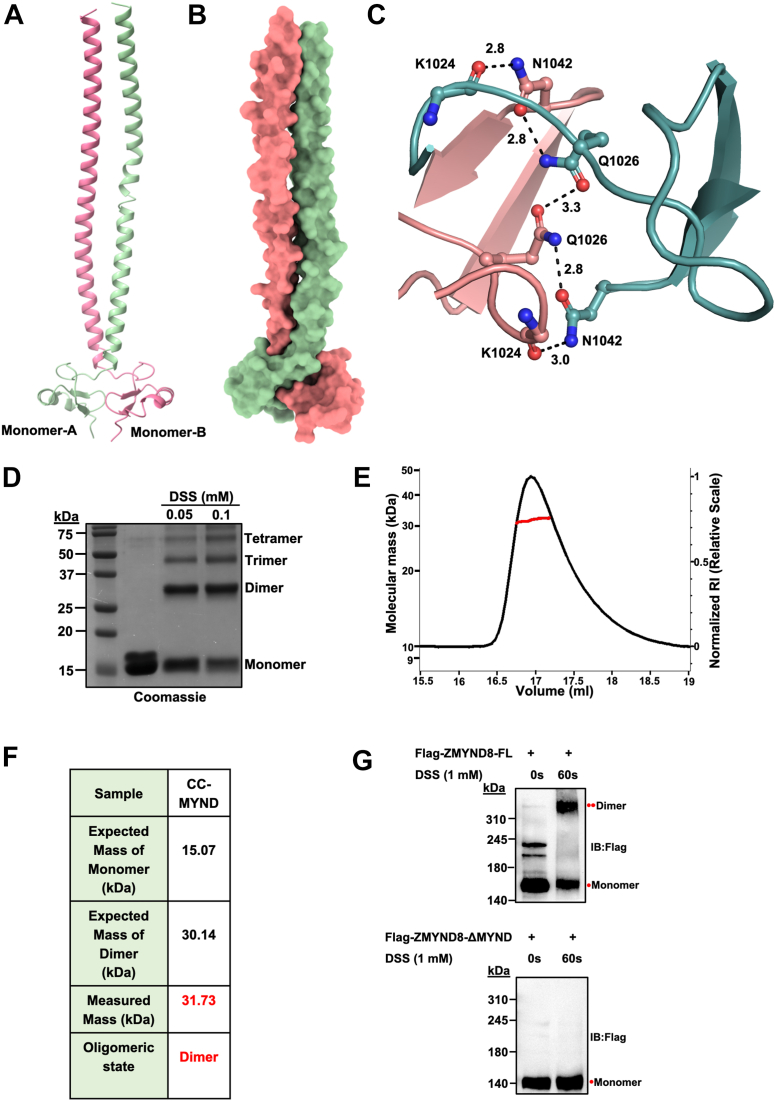
**Analysis of oligomeric state of ZMYND8 CC-MYND.***A*, ribbon and cartoon representation of a ZMYND8 CC-MYND dimer. *B*, surface representation of a ZMYND8 CC-MYND dimer showing extensive surface interaction between the MYND domains of two monomers. *C*, detailed molecular view of the interaction between two MYND domains of a CC-MYND dimer showing extensive interchain hydrogen bond interaction (*black dashed line*) among the residues as indicated. *D*, cross-linked ZMYND8 CC-MYND protein separated on 15% SDS-PAGE gel and protein bands are visualized by Coomassie staining. Different species formed during cross-linking by disuccinimidyl suberate are marked and indicated. *E*, SEC-MALS analysis of ZMYND8 CC-MYND for determination of in-solution oligomeric form. The chromatogram shows the dRI trace (*black line*) plotted over elution volume (ml). The red line indicates the distribution profile of molecular mass of the species eluted under the curve. *F*, summary table of molar mass calculation by SEC–MALS (marked in *red*) and the theoretical molecular weight predictions (marked in *black*) of ZMYND8 CC-MYND domain. *G*, disuccinimidyl suberate mediated *ex vivo* crosslinking assay shows that ZMYND8-FL forms dimers (*upper panel*) whereas ZMYND8-ΔMYND is completely dimer-deficient (*lower panel*). CC-MYND, coiled coil MYND; ZMYND8, zinc finger MYND-type containing eight protein; ZMYND8-FL, full-length ZMYND8.

To fully characterize the quaternary structure and functional assembly state of ZMYND8, we investigated the oligomeric properties of the CC-MYND using complementary crystallographic, biochemical, and biophysical approaches. Crystal structure reveals a dimeric ZMYND8 CC-MYND. Examination of the crystal packing in our ZMYND8 CC-MYND structure revealed a striking dimeric arrangement (Fig. 3*A*). The dimer interface is primarily mediated by interactions between the MYND domains of two monomers, while the CC regions extend away from the dimerization interface. Buried surface area analysis revealed that 5089 Å^2^ was buried at the dimer interface out of a total surface area of 17,246 Å^2^. This substantial buried interface area between the interacting MYND domains indicates energetically favorable dimerization (Fig. 3*B*). To understand the molecular basis of this dimerization, we analyzed the interactions at the dimer interface in detail (Fig. 3*C*). The interface is stabilized by an extensive network of interchain hydrogen bonds. Key residues involved in these interactions are Lys-1024, Asn-1042 and Gln-1026, which are highlighted by ball and stick representation. These residues form a network of interchain contacts with bond distances ranging from 2.8 to 3.3 Å, consistent with strong hydrogen bonding interactions. This extensive hydrogen bonding network suggests that the dimeric assembly is energetically stable and likely functionally relevant.

To validate our crystallographic observations and determine whether the dimeric state persists in solution, we performed an *in vitro* cross-linking assay using disuccinimidyl suberate (DSS) as the cross-linking reagent. SDS-PAGE analysis of cross-linked ZMYND8 CC-MYND samples revealed concentration-dependent formation of covalently linked species (Fig. 3*D*). At increasing DSS concentrations (0.05, 0.1 mM), we observed progressive formation of dimeric, trimeric, and tetrameric species, with the dimeric form being the most prominent cross-linked product. This pattern is consistent with a predominantly dimeric solution state that can assemble into higher-order oligomers under cross-linking conditions.

To definitively establish the solution oligomeric state, we employed size-exclusion chromatography coupled with multi-angle light scattering (SEC-MALS) analysis. The SEC-MALS chromatogram shows a single, well-defined peak with a measured molecular mass of 31.73 kDa (Fig. 3, *E* and *F*). This experimentally determined mass closely matches the calculated dimeric molecular weight (30.14 kDa for the dimer *versus* 15.07 kDa for the monomer), with only 2.6% deviation from the expected dimeric mass. The high precision of this measurement and the absence of significant monomeric or higher-order species confirm that ZMYND8 CC-MYND exists predominantly as a stable homodimer in solution. To extrapolate these findings to cellular context in full-length ZMYND8 proteins, we first performed DSS mediated *ex vivo* crosslinking assay using cellular lysates expressing Flag-ZMYND8-FL or Flag-ZMYND8-ΔMYND. While ZMYND8-FL was able to form significant population of dimers (Fig 3*G*, upper panel), ZMYND8-ΔMYND showed complete absence of dimeric species (Fig 3*G*, lower panel) which confirmed the role of MYND domain in ZMYND8 dimerization. Although ZMYND8 CC-MYND primarily exists as a dimer in solution, examination of the crystallographic asymmetric unit revealed a tetrameric assembly of two dimers (Fig. S3). Interestingly, this tetrameric assembly is not observed in solution (Fig 3*E*).

In summary, our structural, biochemical, and biophysical analyses converge on the conclusion that ZMYND8 CC-MYND forms stable dimers which in turn mediates ZMYND8 dimerization in cells. The crystal structure reveals the molecular details of this dimerization, which is primarily mediated by interactions between the MYND domains. Chemical cross-linking experiments support the existence of dimers and potentially higher-order oligomers in solution, while SEC-MALS analysis confirms that the dimer is the predominant species under physiological-like conditions.

### Molecular characterization of ZMYND8 CC-MYND and GATAD2A interaction

**Figure 4:**
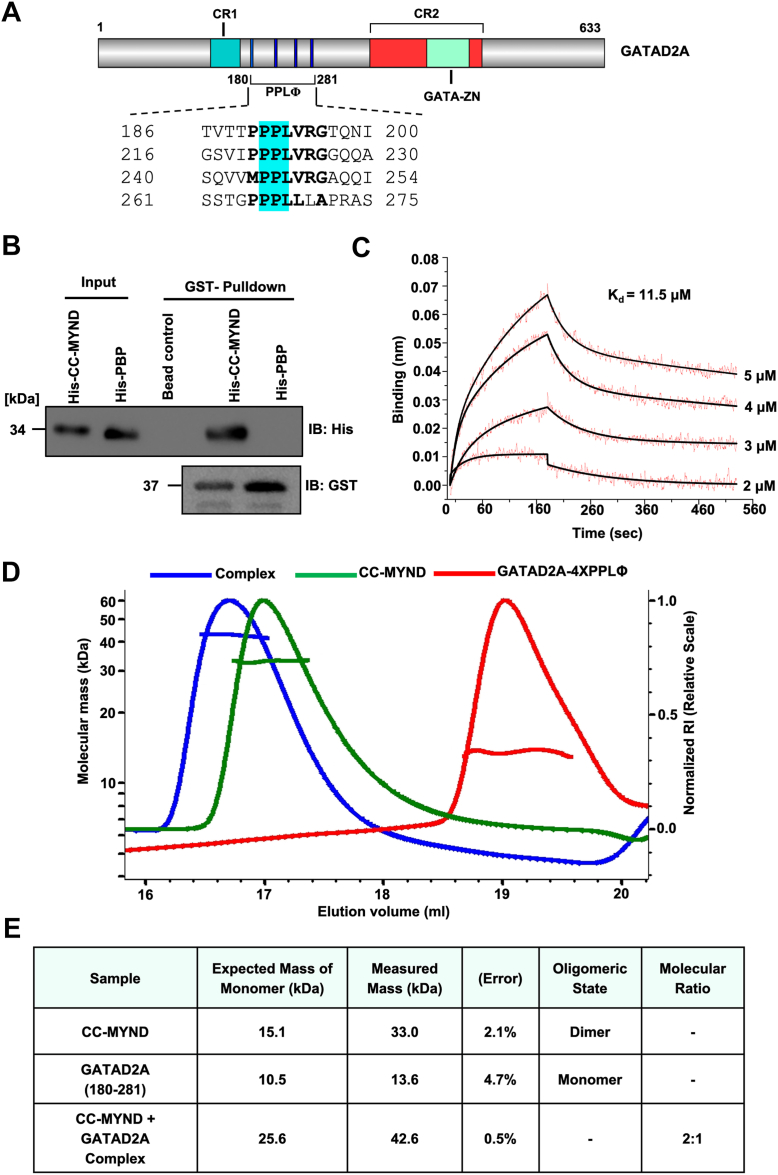
**Molecular characterization of ZMYND8 CC-MYND and GATAD2A interaction.***A*, schematic representation of GATAD2A domain organization showing the N-terminal CR1 domain, central region (residues 180–281) containing four PPPLΦ motifs (where Φ represents hydrophobic residues), and C-terminal CR2 domain with GATA-type zinc finger. Domain boundaries and motif sequences are indicated. The figure was generated using IBS illustrator. *B*, GST pull-down assay between GATAD2A and ZMYND8. GST-GATAD2A (180–281) was used as bait protein and tested for binding to His-tagged ZMYND8 CC-MYND or PBP (Plant Homeodomain-bromodomain-PWWP) domains. Western blot analysis was performed using anti-His and anti-GST antibodies as indicated. Input samples and GST bead controls are shown (C) Quantitative binding analysis between ZMYND8 CC-MYND and GATAD2A (180–281) by biolayer interferometry. Individual curves represent different concentrations as indicated in the figure. *D*, SEC-MALS analysis of complex formation between ZMYND8 CC-MYND and GATAD2A (180–281). The dRI trace over eluted volume for indivudal samples are color coded and indicated and the respective molecular mass distribution of different samples are also shown. *E*, summary table of SEC-MALS results showing expected monomer masses, measured masses, error percentages, oligomeric states, and molecular ratios for individual proteins and the complex. CC-MYND, coiled coil MYND; ZMYND8, zinc finger MYND-type containing eight protein; ZMYND8-FL, full-length ZMYND8.

Given our functional demonstration that ZMYND8 regulates MAPT213 expression through GATAD2A recruitment (Fig. 1), we sought to characterize the direct molecular interaction between these proteins. GATAD2A contains multiple functional domains including an N-terminal CR1 domain, a central region (residues 180–281) containing four interspersed PPPLΦ motifs (where Φ represents a hydrophobic residue), and a C-terminal CR2 domain harboring a GATA-type zinc finger (Fig. 4*A*). The PPPLΦ motifs, located at positions 186 to 200, 216 to 230, 240 to 254, and 261 to 275, are potential protein-protein interaction sites that could mediate binding to ZMYND8.

To determine which domain of ZMYND8 is responsible for GATAD2A(180–281) interaction, we performed GST pull-down experiments using purified recombinant proteins. GST-tagged GATAD2A (180–281) was immobilized and tested for binding to His-tagged ZMYND8 CC-MYND or His-tagged PBP (PHD-bromodomain-PWWP) domains (Fig. 4*B*). Western blot analysis revealed robust binding between GATAD2A (180–281) and ZMYND8 CC-MYND, while no detectable interaction was observed with the PBP domains. This result definitively establishes that the CC-MYND region, rather than the chromatin-binding domains, mediates the specific protein-protein interaction with GATAD2A.

To quantitatively characterize the binding affinity and kinetics of the ZMYND8-GATAD2A interaction, we employed biolayer interferometry. GST-GATAD2A (180–281) was immobilized on biosensor tips, and binding to ZMYND8 CC-MYND was monitored at concentrations ranging from 2 to 5 μM (Fig. 4*C*). The resulting sensorgrams show clear association and dissociation phases, with binding curves that were best fitted using a 2:1 local fitting model. The measured dissociation constant (K_d_= 11.5 μM) indicates a moderately high-affinity interaction that falls within the range typical of physiologically relevant protein-protein interactions involved in transcriptional regulation.

To determine the stoichiometry and solution behavior of the ZMYND8-GATAD2A complex, we performed SEC-MALS analysis. Individual protein samples and their mixture were analyzed to determine accurate molecular weights and complex composition (Fig. 4, *D* and *E*). ZMYND8 CC-MYND alone exhibited a measured molecular mass of 33.0 kDa (calculated monomer mass: 10.5 kDa) (2.1% error), confirming its dimeric state in solution as previously established (Fig. 3). GATAD2A (180–281) showed a measured mass of 13.6 kDa (4.7% error), consistent with a monomeric state (calculated monomer mass: 10.5 kDa). When the two proteins were mixed and analyzed together, the resulting complex exhibited a measured molecular mass of 42.6 kDa (0.5% error). Based on the individual molecular weights and the complex mass, the data are most consistent with a 2:1 stoichiometry, where one ZMYND8 CC-MYND dimer (30.2 kDa) associates with one GATAD2A (180–281) monomers (10.5 kDa), yielding a calculated complex mass of ∼ 40.7 kDa. The formation of a 2:1 complex is consistent with our biolayer interferometry data requiring a 2:1 binding model (Fig. 4*C*). Collectively, these data demonstrate that ZMYND8 recruits GATAD2A through a direct interaction mediated by the CC-MYND domain, forming a stable 2:1 complex.

### Identification of PPPLΦ motif in GATAD2A as the interacting sequence with ZMYND8 MYND domain

**Figure 5:**
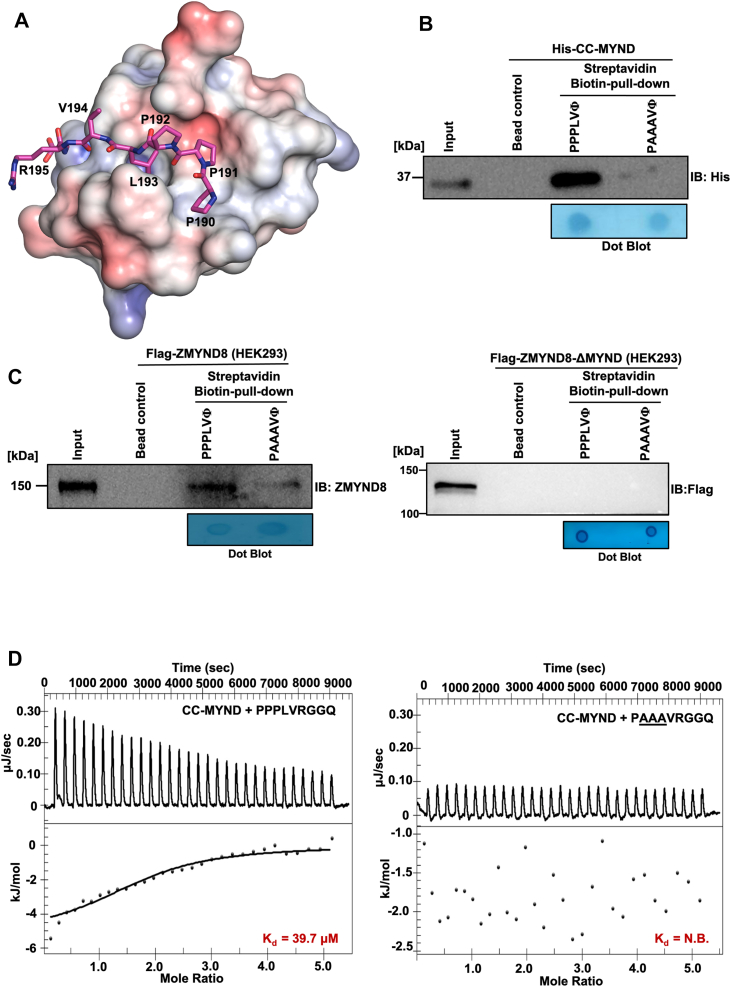
**Identification of PPPLΦ residues in GATAD2A as interacting motif with ZMYND8 CC-MYND domain.***A*, molecular docking model showing the ZMYND8 MYND domain (surface representation) in complex with a GATAD2A peptide containing the PPPLΦ motif. Key residues Pro191, Pro192, and Leu193 (shown in stick representation) are positioned within the MYND domain binding groove. *B*, streptavidin pull-down analysis using biotinylated GATAD2A peptides to assess binding specificity. WT PPPLΦ peptide (PPPLVRGGQ) and mutant PAAAΦ peptide (PAAAVRGGQ) were tested for binding to recombinant His-tagged ZMYND8 CC-MYND. Western blot analysis with anti-His antibody demonstrates specific binding to the WT peptide. Dot blot shows equal peptide loading. Streptavidin beads alone serve as negative control. *C*, Cellular validation of PPPLΦ motif recognition using ZMYND8-FL (*left panel*) and ZMYND8-ΔMYND (*right panel*). FLAG-ZMYND8-FL and Flag-ZMYND8-ΔMYND was overexpressed in HEK293 cells and whole cell lysates were subjected to streptavidin pull-down with biotinylated WT and mutant peptides. Western blot analysis with anti-ZMYND8 and anti-Flag antibody confirms specific binding to the PPPLΦ motif in a cellular context. Corresponding peptide *dot blot* shows equal loading of biotinylated peptides. *D*, ITC analysis of the binding between ZMYND8 CC-MYND and WT (PPPLΦ) & mutated (PAAAΦ) GATAD2A peptide. The titration plot was analyzed and fitted using independent model in NanoAnalyze software. Dissociation constant (K_d_) values are mentioned. ITC, isothermal titration calorimetry; CC-MYND, coiled coil MYND; ZMYND8, zinc finger MYND-type containing eight protein; ZMYND8-FL, full-length ZMYND8.

To elucidate the molecular basis of ZMYND8-GATAD2A recognition and determine the minimal binding determinants, we employed computational docking analysis using the high-resolution ZMYND8 MYND domain crystal structure and GATAD2A peptide sequences containing individual PPPLΦ motifs. Molecular docking studies revealed that the PPPLΦ motif adopts a specific binding mode within a surface groove on the ZMYND8 MYND domain (Fig. 5*A*). The docking model demonstrates that residues Pro-191, Pro-192, and Leu-193 of the GATAD2A peptide make critical contacts within a complementary binding cavity formed by the MYND domain surface. The two consecutive proline residues adopt an extended conformation that positions the hydrophobic leucine residue (Φ) in a hydrophobic pocket, suggesting a structure-based recognition mechanism. To experimentally validate the computational predictions and assess the functional importance of individual residues within the PPPLΦ motif, we performed streptavidin pull-down assays using biotinylated peptides. WT GATAD2A peptides containing the intact PPPLΦ sequence (PPPLVRGGQ) and mutant peptides with proline-to-alanine substitutions (PAAAVRGGQ) were tested for their ability to capture recombinant His-tagged ZMYND8 CC-MYND protein (Fig. 5*B*). Western blot analysis using anti-His antibody revealed robust binding between the WT PPPLΦ peptide and ZMYND8 CC-MYND. In contrast, the mutant PAAAΦ peptide showed dramatically reduced binding, confirming that the consecutive proline residues are critical for ZMYND8 recognition. Dot blot analysis confirmed equal peptide loading, validating the specificity of the observed binding differences.

To determine whether the peptide-based binding specificity is maintained in a more physiologically relevant context, we performed pull-down experiments using full-length ZMYND8 and ZMYND8-ΔMYND expressed in mammalian cells. FLAG-ZMYND8-FL and Flag-ZMYND8-ΔMYND was overexpressed in HEK293 cells, and whole cell lysates were subjected to streptavidin pull-down using biotinylated WT and mutant GATAD2A peptides (Fig. 5*C*). Western blot analysis using anti-ZMYND8 antibody demonstrated that the WT PPPLΦ peptide efficiently captured full-length ZMYND8 from cellular lysates whereas the mutant PAAAΦ peptide showed substantially reduced binding (Fig 5*C*, left panel). Expectedly, the same experiment using Flag-ZMYND8-ΔMYND showed no interaction with both WT PPPLΦ and mutant PAAAΦ peptides (Fig 5*C*, right panel). This confirmed that the PPPLΦ motif recognition specificity observed with isolated domains is preserved in the context of full-length ZMYND8 and in a complex cellular environment. This result validates the physiological relevance of our structural and biochemical findings.

Next, to quantitatively characterize the binding affinity and thermodynamics of the ZMYND8-GATAD2A interaction; we performed ITC experiments. The titration of WT PPPLΦ peptide with ZMYND8 CC-MYND protein gave a dissociation constant (K_d_) value of 39.7 uM indicating a moderately strong interaction (Fig. 5*D*, left panel). We then compared the binding of ZMYND8 CC-MYND to mutated PAAAΦ GATAD2A peptide. The ITC binding isotherm showed absence of any binding interactions which confirmed the critical role of the proline residues in the PPPLΦ motif for ZMYND8 recognition (Fig. 5*D*, right panel). Thus our ITC results provide quantitative support for our structural and biochemical findings, demonstrating the specificity and affinity of the PPPLΦ motif recognition by ZMYND8.

### Identification of key residues in ZMYND8 MYND domain mediating interaction with GATAD2A

**Figure 6:**
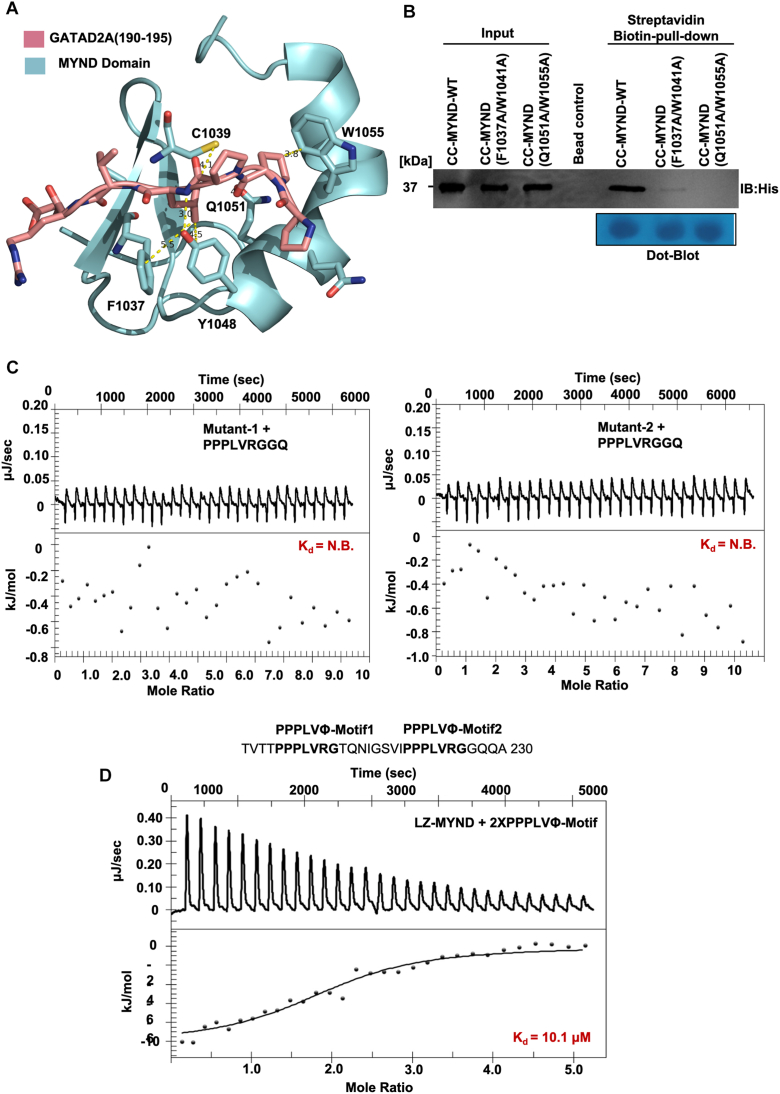
**Identification of residues in ZMYND8 CC-MYND domain involved in its interaction with GATAD2A.***A*, molecular model of the ZMYND8 MYND domain-GATAD2A peptide interface showing critical binding residues. The ZMYND8 MYND domain (cartoon representation) is shown with key interface residues Phe1037, Trp1041, Gln1051, and Trp1055 highlighted in stick representation (shown in *cyan color*). The docked GATAD2A peptide (residues 190–195) containing the PPPLΦ motif is positioned within the binding pocket (shown in *magenta color*). *B*, mutational analysis of ZMYND8 binding interface residues using streptavidin pull-down assays. Biotinylated PPPLΦ peptide was used to capture WT ZMYND8 CC-MYND and two double mutants: Mutant-1 (F1037A/W1041A) and Mutant-2 (Q1051A/W1055A). Western blot analysis with anti-His antibody and *Dot blot* confirms equal peptide loading across samples. *C*, ITC analysis of the GATAD2A PPPLΦ peptide with ZMYND8 CC-MYND Mutant-1 (*left panel*) and Mutant-2 (*right panel*) proteins. *D*, ITC analysis of ZMYND8 CC-MYND binding to a peptide containing two adjacent PPPLΦ motifs corresponding to Motifs 1 and 2 from GATAD2A. Data were analyzed using NanoAnalyze software. Dissociation constant (K_d_) values are mentioned. ITC, isothermal titration calorimetry; CC-MYND, coiled coil MYND; ZMYND8, zinc finger MYND-type containing eight protein; ZMYND8-FL, full-length ZMYND8.

To define the molecular determinants within the ZMYND8 MYND domain responsible for PPPLΦ motif recognition, we performed Molecular docking studies of the ZMYND8-GATAD2A peptide complex using our high-resolution crystal structure. Docking studies with the GATAD2A PPPLΦ peptide (residues 190–195) revealed a well-defined binding interface involving four key residues in the ZMYND8 MYND domain: Ph-1037, Trp-1041, Gln-1051, and Trp-1055 (Fig. 6*A*). These residues form a complementary binding pocket that accommodates the PPPLΦ motif through a combination of hydrophobic and hydrogen bonding interactions. Phe-1037 and Trp-1055 contribute to a hydrophobic surface that interacts with the leucine residue and proline backbone, while Gln-1051 and Tyr-1048 provide additional stabilizing contacts through both hydrophobic interactions and potential hydrogen bonding with the peptide backbone. To experimentally validate the structural predictions and assess the functional contribution of individual residues, we generated ZMYND8 CC-MYND mutants targeting the putative binding interface. Two double mutants were constructed: Mutant-1 (Phe1037Ala/Trp1041Ala) and Mutant-2 (Gln1051Ala/Trp1055Ala), designed to disrupt distinct regions of the binding interface while maintaining overall protein stability. Streptavidin pull-down assays using biotinylated PPPLΦ peptide revealed that both mutants exhibited substantially reduced binding compared to WT ZMYND8 CC-MYND (Fig. 6*B*). Western blot analysis using anti-His antibody demonstrated that Mutant-1 (Phe1037Ala/Trp1041Ala) showed dramatically diminished peptide binding, confirming the critical importance of the hydrophobic residues Phe1037 and Trp1041. Similarly, Mutant-2 (Gln1051Ala/Trp1055Ala) exhibited significantly reduced binding, validating the functional importance of Gln1051 and Trp1055. These results provide direct experimental evidence that all four identified residues contribute to PPPLΦ motif recognition. To quantitatively assess the impact of these mutations on binding affinity, we performed ITC experiments (Fig. 6*C*). The GATAD2A PPPLΦ peptide was titrated against ZMYND8 CC-MYND Mutant-1 (F1037A/W1041A) (Fig 6*C*, left panel) and Mutant-2 (Q1051A/W1055A) (Fig 6*C*, right panel) proteins. Both mutants showed almost no binding affinity compared to the WT ZMYND8 CC-MYND. These ITC results provide quantitative support for the importance of the identified residues in mediating the ZMYND8-GATAD2A interaction. Given that GATAD2A contains four PPPLΦ motifs within its central region (residues 180–281), we investigated whether ZMYND8 could engage multiple motifs simultaneously to achieve enhanced binding affinity. We synthesized a peptide containing two adjacent PPPLΦ motifs (Motif 1 and Motif 2: TVTTPPPLVRGTQNIGSVIPPPLVRGGQQA) corresponding to the natural spacing found in GATAD2A and performed ITC analysis with ZMYND8 CC-MYND (Fig. 6*D*). The dual-motif peptide exhibited significantly enhanced binding affinity (K_d_= 10.1 μM) compared to the single PPPLΦ motif peptide (K_d_= 39.7 μM), representing approximately a 4-fold improvement in binding strength. This binding isotherm suggests cooperative binding mechanisms. The enhanced affinity likely results from avidity effects, where the dimeric ZMYND8 CC-MYND can simultaneously engage multiple PPPLΦ motifs within a single GATAD2A molecule.

## Discussion

This study establishes the molecular mechanism by which ZMYND8 regulates lncRNA MAPT213 expression through MYND domain-mediated recruitment of GATAD2A. Building upon previous observations that ZMYND8 inversely regulates MAPT and MAPT213 expression during neuronal differentiation (1), we now provide structural and biochemical evidence defining how the MYND domain orchestrates this regulatory function through specific protein-protein interactions. Our most significant finding is that ZMYND8 employs a modular mechanism to control MAPT213 transcription through MYND domain-dependent recruitment of the NuRD complex component GATAD2A. The functional importance of the MYND domain is demonstrated by multiple lines of evidence. First, deletion of the MYND domain abolishes ZMYND8's ability to properly regulate both MAPT and MAPT213 expression, despite intact chromatin binding. Second, the MYND domain is essential for GATAD2A interaction both *in vitro* and in cellular contexts. Third, point mutations within the MYND domain binding interface (F1037A/W1041A and Q1051A/W1055A) phenocopy the ΔMYND deletion, failing to recruit GATAD2A to chromatin and losing the ability to regulate MAPT/MAPT213 expression. These results collectively demonstrate that MYND domain integrity is required for ZMYND8-mediated transcriptional regulation at the MAPT locus. The crystal structure of the ZMYND8 CC-MYND domain reveals a modular architecture optimized for both dimerization and protein recruitment. The 2.29 Å resolution structure shows an L-shaped organization with an extended CC region perpendicular to the zinc-coordinating MYND domain. The dimeric arrangement observed both crystallographically and in solution (confirmed by SEC-MALS) suggests that dimerization may enhance the avidity of ZMYND8 for GATAD2A and potentially enable cooperative recruitment of downstream interaction partners. However, we note that the tetrameric assembly observed in the crystallographic asymmetric unit may represent a crystallization artifact rather than a physiologically relevant oligomeric state, and its functional significance requires further investigation in cellular contexts. The structural basis for ZMYND8-GATAD2A recognition involves specific interaction between the MYND domain and PPPLΦ motifs in GATAD2A's central region. Our docking models, validated by mutagenesis and ITC experiments, identify key residues in ZMYND8 that form a complementary binding pocket accommodating the proline-rich motifs. The moderate binding affinity of individual PPPLΦ motifs (K_d_ ∼40 μM) is significantly enhanced when two adjacent motifs are engaged simultaneously (K_d_ ∼10 μM), providing direct evidence for multivalent binding. Given that GATAD2A contains four PPPLΦ motifs within its central region, and our SEC-MALS data indicate a 2:1 stoichiometry (ZMYND8 dimer : GATAD2A monomer), we propose that dimeric ZMYND8 can engage multiple motifs within a single GATAD2A molecule. This multivalent interaction mechanism achieves both specificity and sufficient stability for functional recruitment while maintaining reversibility for dynamic regulation. Our findings provide a structural and mechanistic framework for understanding how ZMYND8 engages GATAD2A through the MYND domain at the MAPT locus. We propose that ZMYND8 homodimers are recruited to specific chromatin sites through recognition of histone modifications by the N-terminal PHD, bromodomain, and PWWP domains, and that once chromatin-bound, the MYND domains engage GATAD2A through multivalent interactions with PPPLΦ motifs. Whether this interaction is sufficient to recruit the full NuRD complex and whether it promotes chromatin remodeling or histone deacetylation at the MAPT213 locus are important questions that remain to be directly addressed.

In summary, this work provides structural and biochemical characterization of the ZMYND8 CC-MYND domain and defines the molecular basis for its interaction with GATAD2A through PPPLΦ motif recognition. The multivalent, avidity-driven binding mechanism identified here offers a framework for understanding how ZMYND8 achieves selectivity for GATAD2A among NuRD complex components, and how this interaction may contribute to transcriptional regulation at the MAPT locus. Broader elucidation of the downstream consequences of this interaction and its generalizability to other ZMYND8 target genes represent key directions for future investigation.

## Experimental procedures

### Cloning and site directed mutagenesis

The CC-MYND region of ZMYND8 encompassing amino acid residues 951 to 1076 was cloned into pETite N-His SUMO expression vector (Lucigen) with an N-Terminal hexa histidine and SUMO tag. PCR amplification of the corresponding nucleotide sequence was done using ZMYND8-FL. Two rounds of PCR were done to include a proteolytic cleavage site (LEVLFQ/GP) for precission protease enzyme between the SUMO and the CC-MYND insert sequence. Human GATAD2A or p66α gene corresponding to amino acid residue 180 to 281 which contains four interspersed PPLΦ motifs (to be referred as GATAD2A-4XPPLΦ hereon) was codon optimized for expression in bacterial system and cloned into pGEX-6p1 vector (GE HealthCare; catalog no.: GE28–9546–48) using BamH1 and XhoI restriction enzymes sites. Mutations in the MYND domain of ZMYND8 (F1037A/W1041A and Q1051A/W1055A) was introduced by site directed mutagenesis using the QuikChange Site-Directed Mutagenesis Kit (Agilent; catalog no.: 200,513) according to the manufacturer's protocol. The pCMV-FLAG mammalian expression vector was used to clone ZMYND8 WT, ΔMYND, and PBP domain-deleted constructs. Cloning procedures utilized the Gateway system (Invitrogen) following manufacturer protocols, and all constructs were sequence-verified.

### Protein expression and purification

ZMYND8 CC-MYND and GATAD2A (180–281) constructs were transformed into *Escherichia coli* BL21C43(DE3) competent cells. Protein purification as described in (33). Briefly, Large-scale cultures (3 L) were grown in LB medium at 37 °C to mid-log phase (OD_600_= 0.6–0.8), then induced with 0.75 mM IPTG for CC-MYND or 0.5 mM IPTG for GATAD2A. For CC-MYND expression, 50 μM ZnCl_2_ was added prior to induction. Cultures were incubated at 18 °C for 16 h with shaking at 180 rpm. Cells were harvested by centrifugation and resuspended in lysis buffer containing 50 mM Tris-HCl (pH 8.0 for CC-MYND, pH 7.5 for GATAD2A), 200 to 250 mM NaCl, 1 mM β-mercaptoethanol, 20 mM imidazole, 2% glycerol, and 0.05% NP-40. Cell lysis was performed by sonication (500 W sonicator, 5-s pulses with 20-s intervals for 10 min total). Lysates were clarified by centrifugation at 12,000 rpm for 60 min at 4 °C and filtered through 0.22 μm membranes.

His-tagged CC-MYND was purified using Ni-NTA agarose resin (30-min binding), followed by extensive washing and on-resin cleavage with PreScission protease for 36 h at 4 °C. GST-tagged GATAD2A was purified using glutathione-agarose resin (2.5-h binding) and similarly cleaved. Final purification was achieved using Superdex 75 or 200 gel filtration chromatography (GE Healthcare) in 20 mM hepes (pH 7.5), 200 mM NaCl. When required, tagged proteins were eluted using imidazole or glutathione gradients followed by desalting. Protein purity was monitored by SDS-PAGE, and purified proteins were concentrated and stored at −80 °C.

### SEC-MALS

Absolute molecular weights were determined using a Superdex 200 10/300 Gl column (GE Healthcare) connected to a Shimadzu HPLC system coupled with a DAWN HELEOS-II multi-angle light scattering detector and Optilab T-rEX differential refractive index detector (Wyatt Technology). Protein samples (200 μL at 2.5–5.0 mg/ml) were pre-centrifuged at high speed to remove aggregates and injected at 0.35 ml/min in running buffer (20 mM Hepes pH 7.5, 200 mM NaCl). System calibration was performed using bovine serum albumin as a standard. Molecular weight distributions were calculated using the Debye fitting method in ASTRA software (Wyatt Technology; https://www.wyatt.com/products/software/astra.html), which determines molar mass from the ratio of light scattering intensity to differential refractive index signals across the elution peak, independent of column calibration standards.

### Crystallization and structure determination

ZMYND8 CC-MYND protein at 10 mg/ml in 20 mM Hepes (pH 7.5), 200 mM NaCl was screened for crystallization using commercial screens and sitting drop vapor diffusion (1:1 protein:precipitant ratio) at 20 °C. Initial needle crystals appeared in 0.75 M ammonium sulfate, 2% PEG 4000, 0.1 M MES (pH 6.5). Crystal morphology was optimized through micro- and macroseeding to obtain diffraction-quality rod-shaped crystals (34). Crystals were cryoprotected with 30% glycerol in mother liquor and flash-frozen in liquid nitrogen. X-ray diffraction data were collected at 100 K using a Bruker D8 Venture diffractometer equipped with a Photon III detector. Data were processed using PROTEUM2 (https://www.brukersupport.com/ProductDetail?uid=dfc44c79-db90-4383-ba26-38f641985c2a) and XDS (https://xds.mr.mpg.de/) software suites (35), with scaling and merging performed using XSCALE (36). The structure was solved by molecular replacement using Phaser in PHENIX suite (37) with an AlphaFold (38) model generated model as search template. Iterative model building and refinement were performed using COOT (39) and PHENIX (40). Solvent accessible surface areas and buried surface areas were computed using AREAMOL (part of the CCP4 suite) (41). Structural representations were generated using PyMOL (Schrödinger, LLC; The PyMOL Molecular Graphics System, Version 2.0; https://www.pymol.org/), and electrostatic surface potentials were calculated using the Adaptive Poisson-Boltzmann Solver (42).

### *In vitro* cross-linking

Proteins were buffer-exchanged into 20 mM Hepes, pH 7.5, 200 mM NaCl. Cross-linking was performed using DSS (Thermo Fisher Scientific) at concentrations of 0.05 to 0.25 mM. CC-MYND (1 mg/ml) was incubated with cross-linkers for 30 min at room temperature, and reactions were quenched with 50 mM Tris-Cl, pH 7.5. Samples were resolved on 15% SDS-PAGE and stained with Coomassie blue.

### GST-pull down assays

His-tagged CC-MYND or His-PBP (PHD-Bromo-PWWP) of ZMYND8 was mixed with GST-GATAD2A-4XPPLΦ in pull-down buffer (50 mM Tris-Cl, pH 7.5, 200 mM NaCl, 1 mM PMSF, 0.05% NP-40) and incubated overnight. GST-agarose beads were added, and binding was allowed for 2 h at 4 °C. Beads were washed, and bound proteins were eluted and analyzed by SDS-PAGE and Western blotting using anti-His and anti-GST antibodies.

### Streptavidin pull-down assay

Biotinylated peptides (WT: PPPLVRGGQ; mutant: PAAAVRGGQ) were incubated with His-CC-MYND or cell lysates from ZMYND8-transfected HEK293 cells. Streptavidin-agarose beads were added, and binding was allowed for 2 h. Beads were washed, and samples were analyzed by SDS-PAGE and Western blotting using anti-His or anti-ZMYND8 antibodies.

### Biolayer interferometry

Biolayer interferometry was performed on an Octet 96 system (PAL ForteBio). GST-GATAD2A-4XPPLΦ was immobilized on anti-GST sensors, and interactions with CC-MYND were measured. Data were analyzed using Octet Data Analysis software (https://www.sartorius.com/en/products/biolayer-interferometry/octet-systems-software), and dissociation constants were calculated using a 2:1 local fitting model.

### ITC

ITC experiments were performed using a NanoITC instrument (TA Instruments). CC-MYND and GATAD2A peptides were dialyzed into 20 mM Hepes, pH 7.5, 200 mM NaCl. Titrations were performed at 25 °C, and data were analyzed using NanoAnalyze software (https://www.tainstruments.com/sw/nano_analyze.html). Binding constants and thermodynamic parameters were calculated.

### Docking

The CABS-dock web server was employed to model the interaction between the ZMYND8-MYND domain and GATAD2A (43). The crystal structure of ZMYND8CC-MYND served as the starting coordinates for the protein domain, while the coordinates of GATAD2A residues 190 to 195 were generated from the amino acid sequence. The resulting docked models were visualized using PyMOL (Schrödinger, LLC; The PyMOL Molecular Graphics System, Version 2.0).

### Cell culture and treatments

SHSY5Y cells are cultured in DMEM F12 medium (Gibco) and supplemented with 10% FBS (Gibco) and 1% Anti-bacterial and antimycotic solution (Gibco). Cells were maintained in humidified incubator 5% CO_2_ incubator.

### Overexpression and shRNA transfection

ZMYND8 full-length (FL), ΔMYND, and PBP domain-deleted constructs were cloned into pCMV-FLAG and transfected into SHSY5Y cells using Lipofectamine 2000 (Invitrogen). ZMYND8 shRNA (Santa Cruz Biotechnology, catalog no. sc-76337-SH) was cloned into the pLKO1 vector for knockdown experiments.

### Western blotting

Whole-cell lysates were prepared using RIPA buffer (20 mM Tris pH 8.0, 150 mM NaCl, 0.1% SDS,0.5% Sodium deoxycholate, 1% NP-40, 1 mM EDTA), and protein concentrations were determined by the Bradford method. Samples were resolved on SDS-PAGE, transferred to nitrocellulose membranes, and probed with primary and secondary antibodies.

### Co-immunoprecipitation

Co-IP was performed using a previous protocol, as mentioned elsewhere [16]. Briefly, cells were scrapped off and harvested and lysed using RIPA buffer (50 mM Tris pH 8, 150 mM NaCl, 0.1% SDS, 0.5% Sodium deoxycholate, 1% NP-40, 1 mM EDTA and complete protease inhibitor cocktail was supplemented. Subsequently lysates were sonicated at 30 Amplitude and 0.5 cycle frequency for 2 min. Subsequently, the cell lysate was centrifuged at 13,000 rpm for 10 min. Pre-clearing with normal sheep serum was done to avoid nonspecific protein-protein interaction; immunoprecipitation was done using FLAG-beads blocked in 5% BSA (Thermo Fisher Scientific) for 3 h. Subsequently, the drawn beads were washed three times with RIPA buffer to remove the nonspecific interactions. Western blotting was done for the analysis of immunoprecipitation.

### DSS mediated *ex vivo* crosslinking

HEK293T cell expressing Flag-tagged ZMYND8-FL or ZMYND8-ΔMYND were lysed in ice using crosslinking lysis buffer (20 mM Hepes pH 7.4, 150 mM NaCl, 2 mM MgCl_2_, 10% glycerol, 0.75% NP-40, 1 mM PMSF, 125U/ml Benzonase Endonuclease). Following lysis, the lysate was centrifuged at 16,000*g* and the supernatant was collected. 40 μg lysate was taken and crosslinking was performed using DSS such that the final concentration of DSS in the reaction volume (20 μl) was 1 mM. The reaction was performed in 30 °C for 1 min, followed by quenching with 1M Tris-base pH 8.0. The samples were run on SDS-PAGE and subsequent western blotting was performed to analyze the extent of cross-linking.

### Quantitative RT-PCR and chromatin immunoprecipitation

Total RNA was extracted using TRIzol reagent (Invitrogen) and reverse-transcribed (1 μg RNA) using the Verso cDNA Synthesis Kit (Thermo Fisher Scientific). Quantitative RT-PCR was performed using SYBR Green Mastermix (Bio-Rad) on a StepONE Plus system with standard cycling conditions (94 °C denaturation, primer-specific annealing, 72 °C extension). Data represent three independent experiments with technical triplicates. Cells were cross-linked with 1% formaldehyde for 10 min at room temperature, quenched with 125 mM glycine, and lysed sequentially in Farnham's lysis buffer (5 mM PIPES pH 8.0, 0.5% NP-40, 85 mM KCl) and nuclear lysis buffer (50 mM Tris-HCl pH 8.0, 1% SDS, 10 mM EDTA). Chromatin was sonicated and immunoprecipitated using FLAG-M2 beads (Sigma) blocked with 5% BSA for 3 h at 4 °C. Beads were washed sequentially with RIPA, high-salt (500 mM NaCl), LiCl, and TE buffers. Cross-links were reversed by heating at 65 °C overnight, followed by RNase A and Proteinase K treatment. DNA was purified by phenol-chloroform extraction and ethanol precipitation. ChIP enrichment was quantified by qPCR using gene-specific primers. Data represent three independent experiments with technical triplicates.

### Statistical analysis

All ChIP-qPCR and qRT-PCR experiments were performed with three technical replicates and repeated independently a minimum of three times. Data are presented as bar graphs with error bars representing the ±SD. Statistical significance was determined by one-way ANOVA using GraphPad Prism (version 10.2; GraphPad Software Inc.; https://www.graphpad.com/). *p*-values, ∗*p*≤ 0.05; ∗∗*p*≤ 0.001; ∗∗∗*p*≤ 0.001 were considered significant.

## Data availability

Atomic coordinates and structure factors of the crystal structure presented in this article have been deposited with the Protein Data Bank under accession number 9WV4.

## Supporting information

This article contains supporting information.

## Conflict of interest

The authors declare that they have no conflicts of interest with the contents of this article.
