# Supplementary material for: The chromatin reader ZMYND8 recruits the NuRD component GATAD2A through its MYND domain to regulate MAPT213 long noncoding RNA transcription
Source: J Biol Chem. 2026 Apr 16;302(6):111463. doi: 10.1016/j.jbc.2026.111463 (PMC13196413; doi:10.1016/j.jbc.2026.111463)
Supplement: Supporting Information [file mmc1.pptx]

## Slide 1
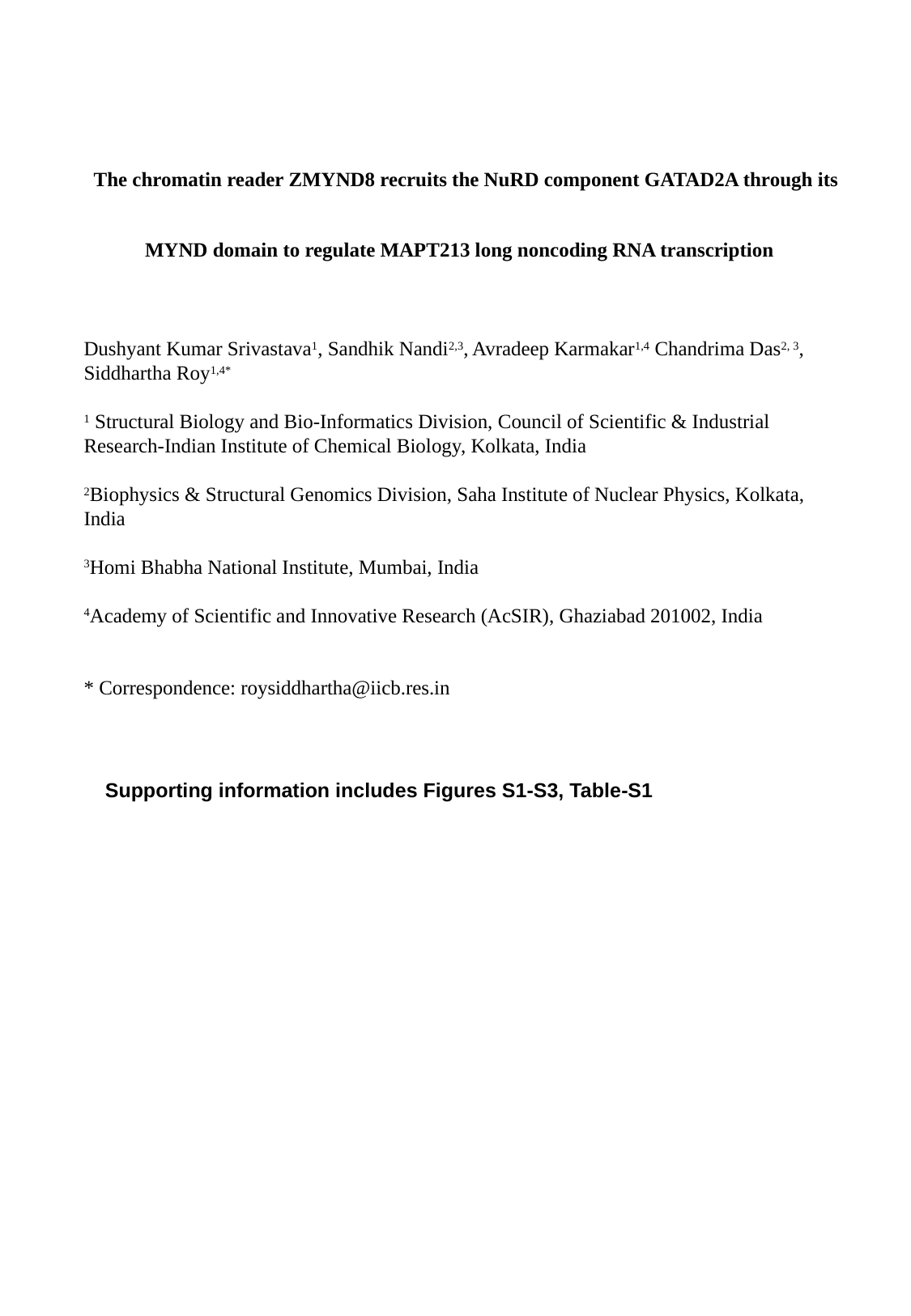

The chromatin reader ZMYND8 recruits the NuRD component GATAD2A through its MYND domain to regulate MAPT213 long noncoding RNA transcription
Dushyant Kumar Srivastava1, Sandhik Nandi2,3, Avradeep Karmakar1,4 Chandrima Das2, 3, Siddhartha Roy1,4*
1 Structural Biology and Bio-Informatics Division, Council of Scientific & Industrial Research-Indian Institute of Chemical Biology, Kolkata, India
2Biophysics & Structural Genomics Division, Saha Institute of Nuclear Physics, Kolkata, India
3Homi Bhabha National Institute, Mumbai, India
4Academy of Scientific and Innovative Research (AcSIR), Ghaziabad 201002, India
* Correspondence: roysiddhartha@iicb.res.in
Supporting information includes Figures S1-S3, Table-S1

## Slide 2
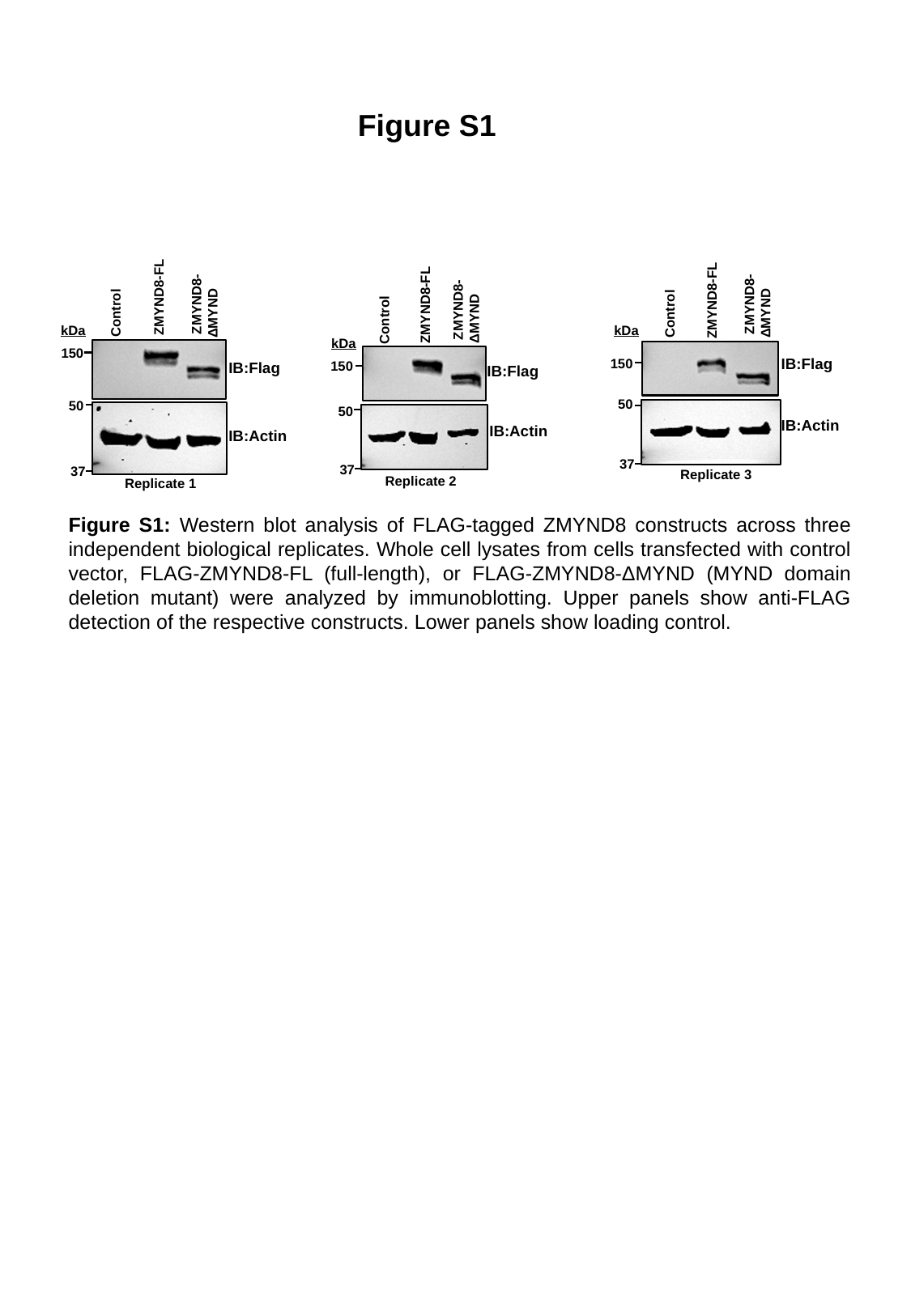

Figure S1
 ZMYND8-ΔMYND
 ZMYND8-FL
Control
kDa
150
50
37
Replicate 1
 ZMYND8-ΔMYND
 ZMYND8-FL
Control
kDa
150
50
37
Replicate 3
 ZMYND8-ΔMYND
 ZMYND8-FL
Control
kDa
150
50
37
Replicate 2
IB:Flag
IB:Flag
IB:Flag
IB:Actin
IB:Actin
IB:Actin
Figure S1: Western blot analysis of FLAG-tagged ZMYND8 constructs across three independent biological replicates. Whole cell lysates from cells transfected with control vector, FLAG-ZMYND8-FL (full-length), or FLAG-ZMYND8-ΔMYND (MYND domain deletion mutant) were analyzed by immunoblotting. Upper panels show anti-FLAG detection of the respective constructs. Lower panels show loading control.

## Slide 3
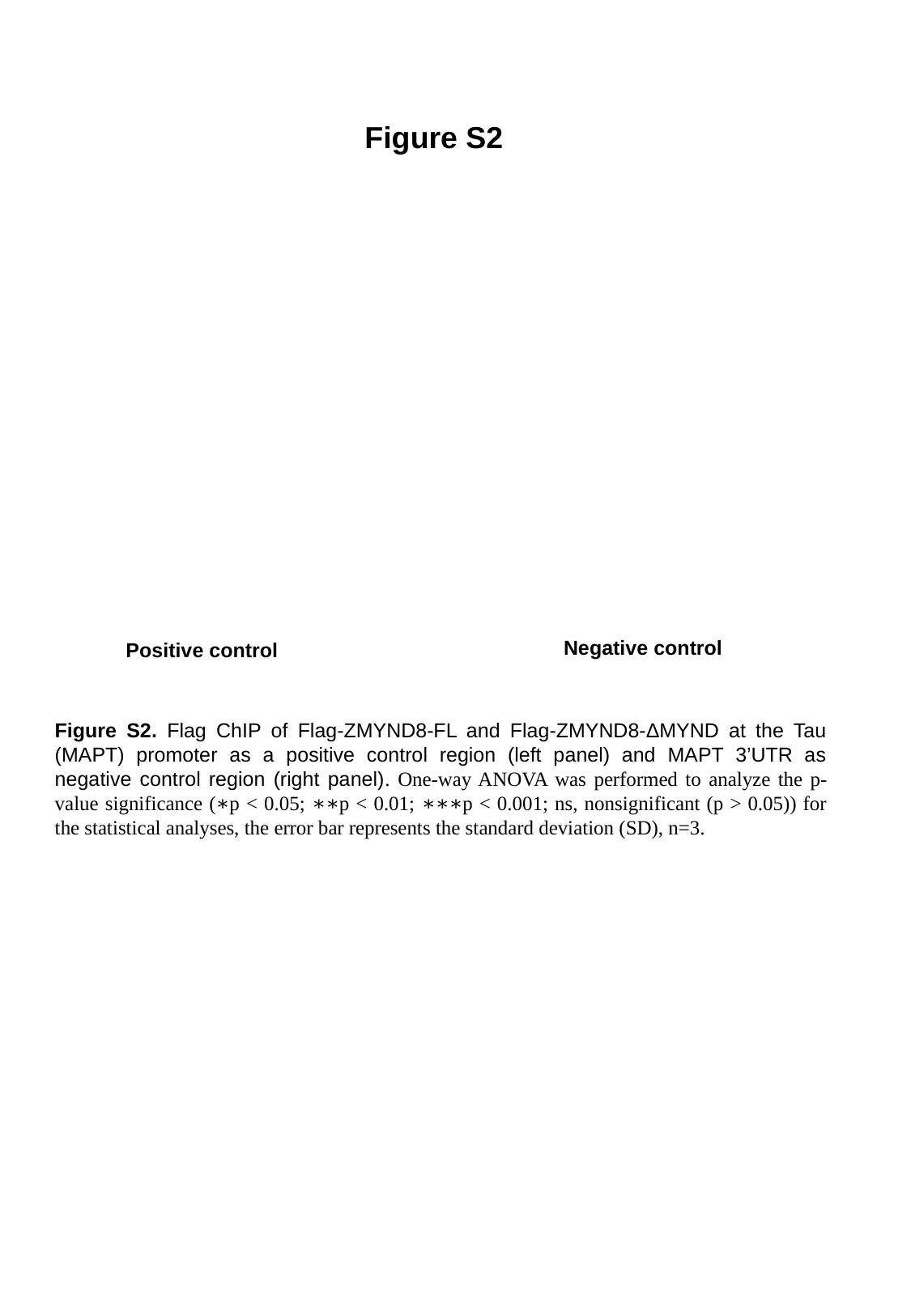

Figure S2
Negative control
Positive control
Figure S2. Flag ChIP of Flag-ZMYND8-FL and Flag-ZMYND8-ΔMYND at the Tau (MAPT) promoter as a positive control region (left panel) and MAPT 3’UTR as negative control region (right panel). One-way ANOVA was performed to analyze the p-value significance (∗p < 0.05; ∗∗p < 0.01; ∗∗∗p < 0.001; ns, nonsignificant (p > 0.05)) for the statistical analyses, the error bar represents the standard deviation (SD), n=3.

## Slide 4
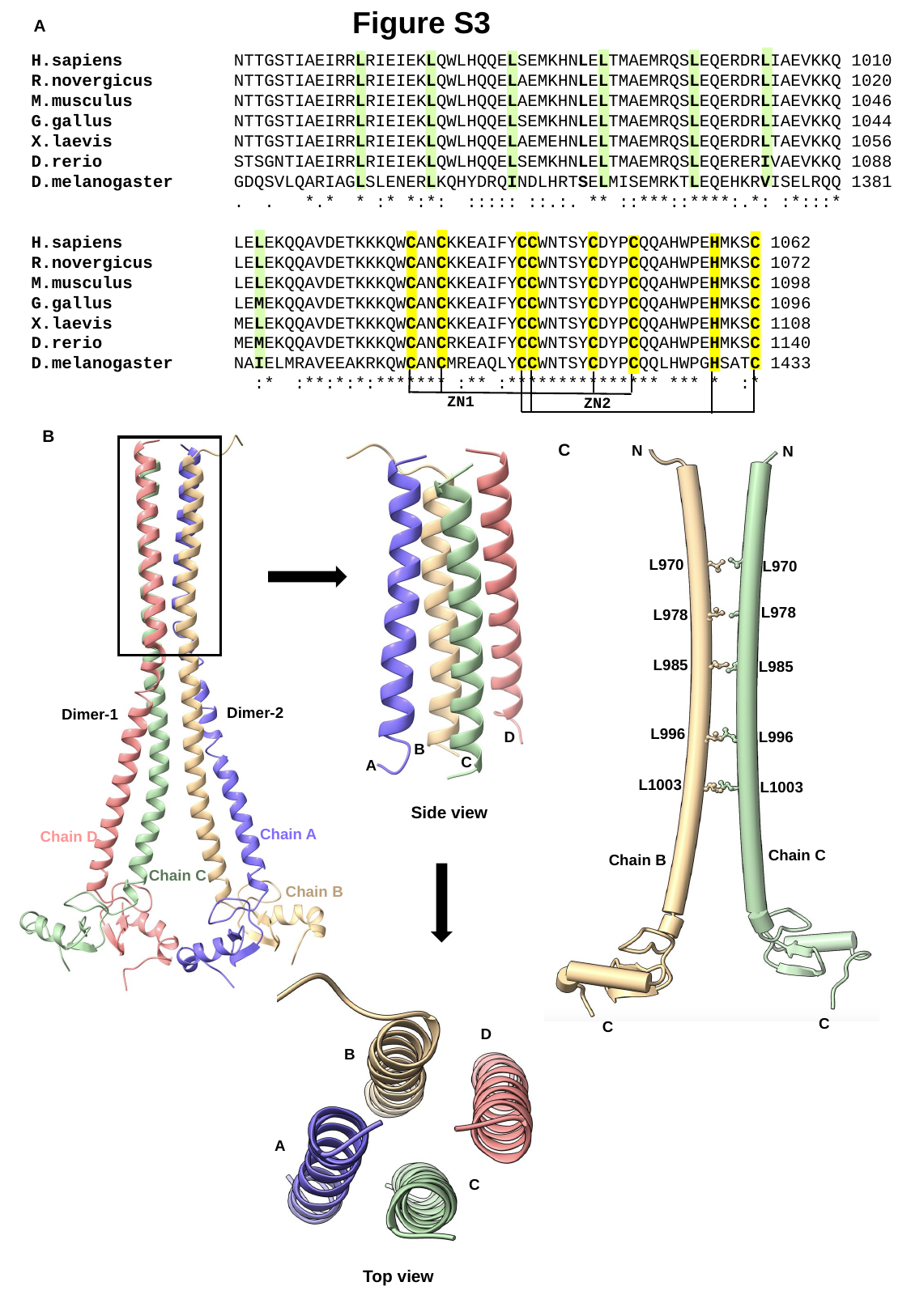

Figure S3
A
H.sapiens           NTTGSTIAEIRRLRIEIEKLQWLHQQELSEMKHNLELTMAEMRQSLEQERDRLIAEVKKQ 1010
R.novergicus        NTTGSTIAEIRRLRIEIEKLQWLHQQELAEMKHNLELTMAEMRQSLEQERDRLIAEVKKQ 1020
M.musculus          NTTGSTIAEIRRLRIEIEKLQWLHQQELAEMKHNLELTMAEMRQSLEQERDRLIAEVKKQ 1046
G.gallus            NTTGSTIAEIRRLRIEIEKLQWLHQQELSEMKHNLELTMAEMRQSLEQERDRLIAEVKKQ 1044
X.laevis            NTTGSTIAEIRRLRIEIEKLQWLHQQELAEMEHNLELTMAEMRQSLEQERDRLTAEVKKQ 1056
D.rerio             STSGNTIAEIRRLRIEIEKLQWLHQQELSEMKHNLELTMAEMRQSLEQERERIVAEVKKQ 1088
D.melanogaster      GDQSVLQARIAGLSLENERLKQHYDRQINDLHRTSELMISEMRKTLEQEHKRVISELRQQ 1381
                    .  .   *.*  * :* *:*:  ::::: ::.:. ** ::***::****:.*: :*:::*
H.sapiens           LELEKQQAVDETKKKQWCANCKKEAIFYCCWNTSYCDYPCQQAHWPEHMKSC 1062
R.novergicus        LELEKQQAVDETKKKQWCANCKKEAIFYCCWNTSYCDYPCQQAHWPEHMKSC 1072
M.musculus          LELEKQQAVDETKKKQWCANCKKEAIFYCCWNTSYCDYPCQQAHWPEHMKSC 1098
G.gallus            LEMEKQQAVDETKKKQWCANCKKEAIFYCCWNTSYCDYPCQQAHWPEHMKSC 1096
X.laevis            MELEKQQAVDETKKKQWCANCKKEAIFYCCWNTSYCDYPCQQAHWPEHMKSC 1108
D.rerio             MEMEKQQAVDETKKKQWCANCRKEAIFYCCWNTSYCDYPCQQAHWPEHMKSC 1140
D.melanogaster      NAIELMRAVEEAKRKQWCANCMREAQLYCCWNTSYCDYPCQQLHWPGHSATC 1433
                      :*  :**:*:*:******* :** :*************** *** *  :*
ZN1
ZN2
B
Dimer-2
Dimer-1
Chain A
Chain D
Chain C
Chain B
D
B
C
A
Side view
D
B
A
C
Top view
C
N
N
L970
L970
L978
L978
L985
L985
L996
L996
L1003
L1003
C
C
Chain C
Chain B

## Slide 5
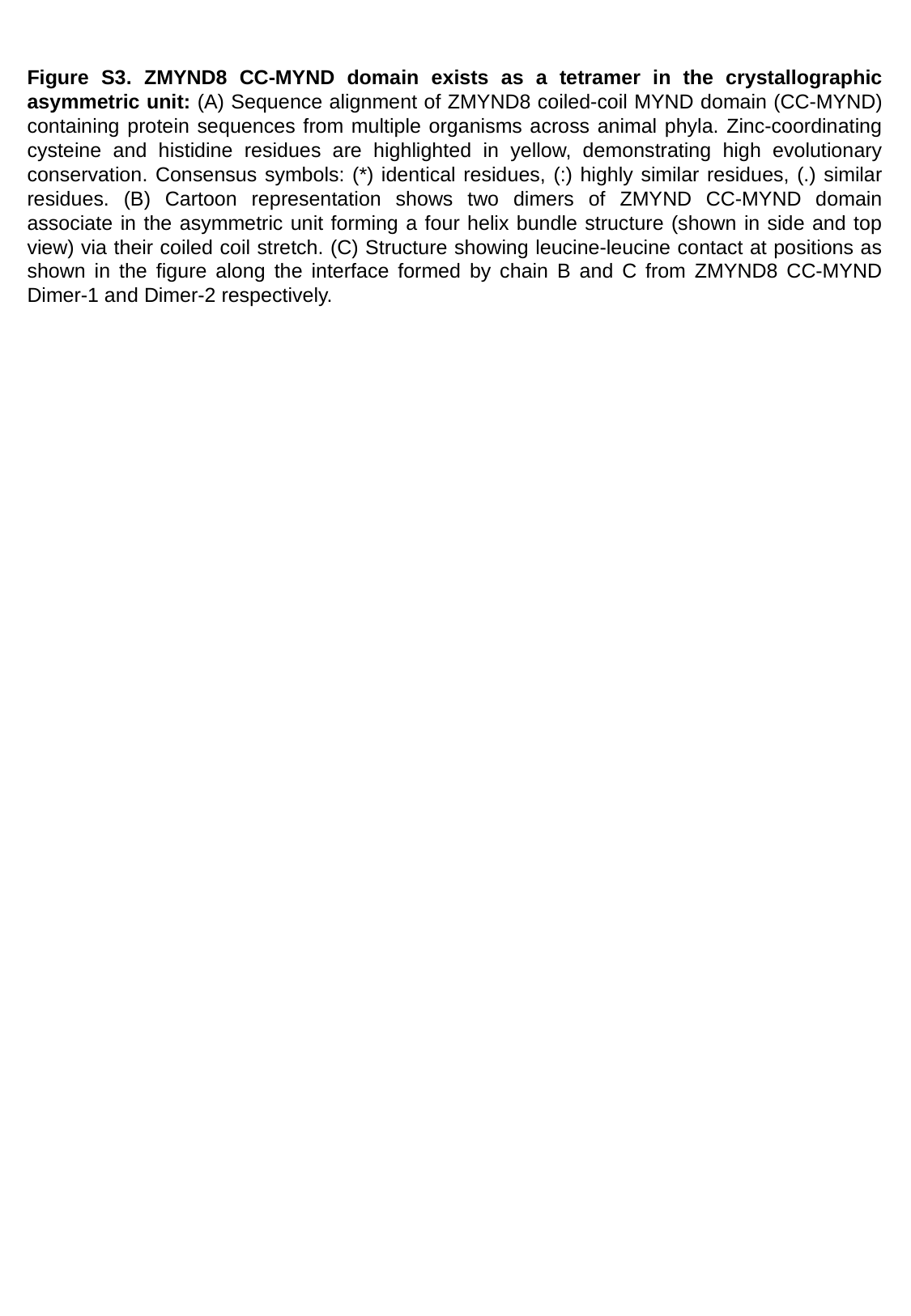

Figure S3. ZMYND8 CC-MYND domain exists as a tetramer in the crystallographic asymmetric unit: (A) Sequence alignment of ZMYND8 coiled-coil MYND domain (CC-MYND) containing protein sequences from multiple organisms across animal phyla. Zinc-coordinating cysteine and histidine residues are highlighted in yellow, demonstrating high evolutionary conservation. Consensus symbols: (*) identical residues, (:) highly similar residues, (.) similar residues. (B) Cartoon representation shows two dimers of ZMYND CC-MYND domain associate in the asymmetric unit forming a four helix bundle structure (shown in side and top view) via their coiled coil stretch. (C) Structure showing leucine-leucine contact at positions as shown in the figure along the interface formed by chain B and C from ZMYND8 CC-MYND Dimer-1 and Dimer-2 respectively.

## Slide 6
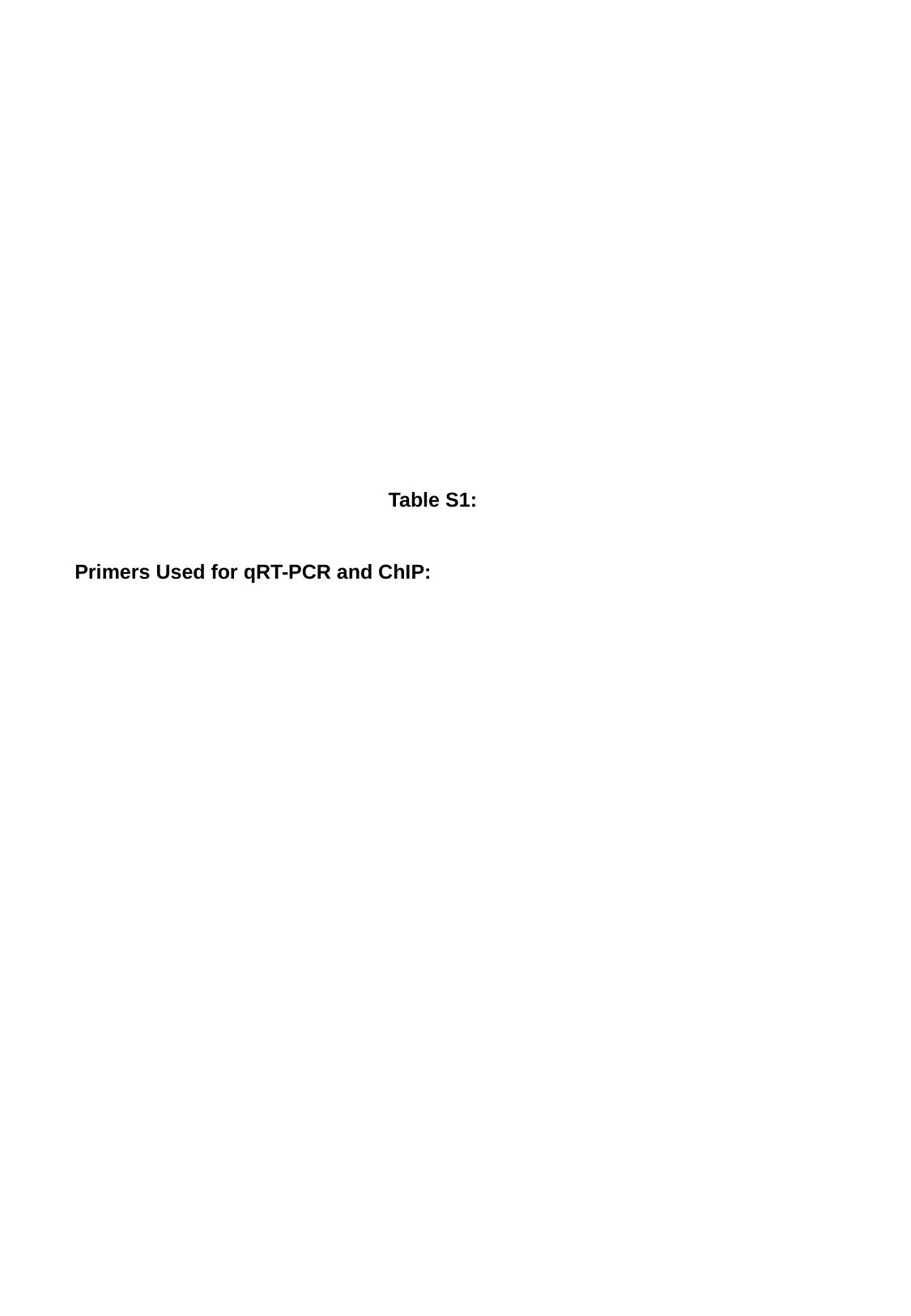

Table S1:
Primers Used for qRT-PCR and ChIP:
